# Effects of urban fine particulate matter and ozone on HDL functionality

**DOI:** 10.1186/s12989-016-0139-3

**Published:** 2016-05-24

**Authors:** Gajalakshmi Ramanathan, Fen Yin, Mary Speck, Chi-hong Tseng, Jeffrey R. Brook, Frances Silverman, Bruce Urch, Robert D. Brook, Jesus A. Araujo

**Affiliations:** 1 Division of Cardiology, Department of Medicine, David Geffen School of Medicine, University of California, Los Angeles, 10833 Le Conte Avenue, CHS 43-264, P.O. Box 951679, Los Angeles, CA 90095 USA; 2 Department of Environmental Health Sciences, Fielding School of Public Health, University of California, Los Angeles, USA; 3 Molecular Biology Institute, University of California, Los Angeles, CA USA; 4 Environment Canada, Toronto, ON Canada; 5 Southern Ontario Centre for Atmospheric Aerosol Research (SOCAAR), Toronto, ON Canada; 6 Division of Occupational and Environmental Health, Dalla Lana School of Public Health, University of Toronto, Toronto, ON Canada; 7 Department of Medicine, Faculty of Medicine, University of Toronto, Toronto, ON Canada; 8 Li Ka Shing Knowledge Institute, St Michael’s Hospital, Toronto, ON Canada; 9 Division of Cardiovascular Medicine, University of Michigan, Ann Arbor, MI USA

**Keywords:** High density lipoprotein (HDL), Air pollution, HDL oxidant index (HOI), HDL function, Fine particulate matter, Cardiovascular, Ozone

## Abstract

**Background:**

Exposures to ambient particulate matter (PM) are associated with increased morbidity and mortality. PM_2.5_ (<2.5 μm) and ozone exposures have been shown to associate with carotid intima media thickness in humans. Animal studies support a causal relationship between air pollution and atherosclerosis and identified adverse PM effects on HDL functionality.

We aimed to determine whether brief exposures to PM_2.5_ and/or ozone could induce effects on HDL anti-oxidant and anti-inflammatory capacity in humans.

**Methods:**

Subjects were exposed to fine concentrated ambient fine particles (CAP) with PM_2.5_ targeted at 150 μg/m^3^, ozone targeted at 240 μg/m^3^ *(120 ppb)*, PM_2.5_ plus ozone targeted at similar concentrations, and filtered air (FA) for 2 h, on 4 different occasions, at least two weeks apart, in a randomized, crossover study. Blood was obtained before exposures (baseline), 1 h after and 20 h after exposures. Plasma HDL anti-oxidant/anti-inflammatory capacity and paraoxonase activity were determined. HDL anti-oxidant/anti-inflammatory capacity was assessed by a cell-free fluorescent assay and expressed in units of a HDL oxidant index (HOI). Changes in HOI (ΔHOI) were calculated as the difference in HOI from baseline to 1 h after or 20 h after exposures.

**Results:**

There was a trend towards bigger ΔHOI between PM_2.5_ and FA 1 h after exposures (*p* = 0.18) but not 20 h after. This trend became significant (*p* <0.05) when baseline HOI was lower (<1.5 or <2.0), indicating decreased HDL anti-oxidant/anti-inflammatory capacity shortly after the exposures. There were no significant effects of ozone alone or in combination with PM_2.5_ on the change in HOI at both time points. The change in HOI due to PM_2.5_ showed a positive trend with particle mass concentration (*p* = 0.078) and significantly associated with the slope of systolic blood pressure during exposures (*p* = 0.005).

**Conclusions:**

Brief exposures to concentrated PM_2.5_ elicited swift effects on HDL anti-oxidant/anti-inflammatory functionality, which could indicate a potential mechanism for how particulate air pollution induces harmful cardiovascular effects.

**Electronic supplementary material:**

The online version of this article (doi:10.1186/s12989-016-0139-3) contains supplementary material, which is available to authorized users.

## Background

Extensive epidemiological evidence demonstrates that air pollution causes adverse health effects leading to increased cardiovascular (CV) and cerebrovascular morbidity and mortality, especially of ischemic nature [1, 2]. Air pollution is a heterogeneous mixture of particulate matter (PM) and gases continuously interacting with each other [1]. Exposure to ambient PM_2.5_ (PM <2.5 μm) has been associated with the development of atherosclerosis [3–6] and the rate of carotid intima-media thickness (CIMT) progression over time [7, 8]. Similarly, sub-chronic exposure studies in animals have shown that apolipoprotein E (ApoE) null mice exposed to PM at ambient levels [9, 10], concentrated PM_2.5_ [11, 12], ultrafine particles (UFP) [13], and diesel exhaust emissions [14], show accelerated atherosclerosis. ApoE null [15] and low density lipoprotein-receptor (LDL-R) null mice [16] exposed to intratracheal particulate, devoid of gases, also display increased severity and complexity of atherosclerotic plaques. However, some studies have shown that PM-filtered mixed vehicular and diesel exhaust emissions induce increased inflammation in the aorta, and altered composition of atherosclerotic plaques in hyperlipidemic mice [17, 18]. In addition, pulmonary exposure to ozone has been shown to promote vascular dysfunction and atherogenesis in ApoE^−/−^ mice [19], and ambient levels of ozone have been associated with CIMT in healthy young adults [20], suggesting that ozone could also promote atherosclerosis. However, the mechanisms for PM and/or ozone-induced CV toxicity are still poorly understood and there is a lack of very much needed, sensitive and reliable biomarkers that could help in identifying CV effects induced by air pollutants.

Risk for CV diseases is increased by a proatherogenic lipid profile, consisting of elevated levels of low-density lipoprotein (LDL) and triglycerides or low levels of high–density lipoprotein cholesterol (HDL-c) [21]. A few studies have shown that ambient air pollutant exposure could induce modest effects on the levels of plasma lipids [22]. Thus, LDL-R null mice exposed to re-aerosolized ultrafine particles (UFP) displayed decreased HDL-c [23]. However, we have shown that concentrated ambient particles (CAPs) in the ultrafine-size range enhanced atherosclerotic lesions in ApoE null mice without affecting HDL levels, but altering its capacity to inhibit inflammation [13]. Likewise, both diesel exhaust and UFP induce dysfunctional HDL that is prooxidant and proinflammatory in ApoE null [24] and LDL-R null mice [23].

Changes in HDL function could be as or even more important adverse health effects than changes in quantitative lipoprotein levels. HDL’s impaired cholesterol efflux capacity is inversely correlated with CIMT and the development of coronary artery disease (CAD), independent of HDL-c levels [25], and subjects with acute coronary syndrome or ischemic cardiomyopathy displayed impaired HDL antioxidant/anti-inflammatory function compared to healthy controls [26, 27]. Importantly, changes in HDL functionality could occur quite rapidly [28–30], last for a few days in reversible disorders such as acute infections [30] or persist for a long time, in subjects with inflammatory disorders such as rheumatoid arthritis [31], systemic lupus erythematosus [32] or CAD [26]. In the present study, we aimed to determine whether brief 2-hour long exposures to PM and/or ozone are capable of inducing adverse changes in HDL functionality in human subjects. In addition, we tested the notion that PM and ozone could exert additive or synergistic effects on HDL functionality.

## Results

### Subject characteristics, exposures and HDL oxidant index

**Fig. 1:**
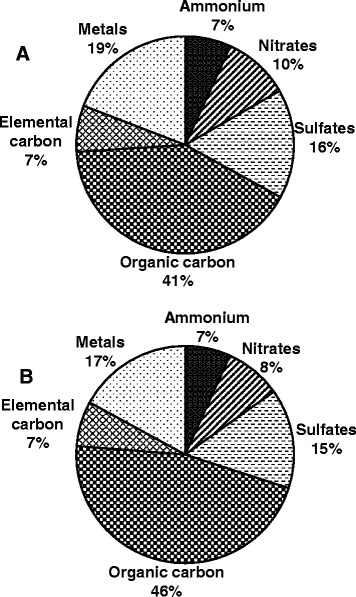
Particle chemical composition of (**a**) PM_2.5_ and (**b**) PM_2.5_ + Ozone exposures. Both PM_2.5_ and PM_2.5_ + Ozone exposure aerosols had similar particle chemical composition, which was performed as described in Methods

We studied plasma samples from subjects participating in the Toronto component of the Clean Air Research Center project, where the effects of PM_2.5_ and/or ozone on blood pressure and vascular function have been reported [33]. Subjects were exposed to filtered air, concentrated PM_2.5_, ozone, or PM_2.5_ + ozone, for 2 h (Additional file 1: Figure S1). They displayed a normal range of lipid and glucose levels representing a healthy cohort (Table 1). The average concentrations of PM_2.5_ and ozone were 149 μg/m^3^ and 221 μg/m^3^ (111 ppb), respectively, in the PM_2.5_ and ozone individual exposures, and 132 μg/m^3^ and 216 μg/m^3^ (109 ppb) in the combined PM_2.5_ + ozone exposures [33] (Table 2). PM chemical composition was rich in organic carbon and similar in both the fine CAP and ozone aerosols (Fig. 1). Plasma HDL anti-oxidant/anti-inflammatory capacity was assessed by a DCF-based cell free assay, and expressed in units of a HDL oxidant index (HOI), as described in the Methods. This assay exhibited a high inter-assay and inter-user reproducibility as indicated in previous reports [34, 35] and shown in Additional file 1: Figure S2. Blood collection is at times accompanied with some degree of hemolysis, which leads to release of hemoglobin with the potential to alter redox chemistry and interfere in our assay. The degree of hemolysis can vary from very low, when it is imperceptible to the eye, to gross red discoloration of the plasma. We estimated the degree of hemolysis by spectrophotometry at a wavelength of 410 nm, where hemoglobin shows an absorption peak [36]. Indeed, we noted there was a significant positive association between HOI and optimal density (OD) values (*r* = 0.545, p < 0.0001, Additional file 1: Figure S3A), likely due to the presence of higher levels of heme and redox-active iron, which could promote redox reactions and artifactual increase of the HOI. However, we did not observe any association when OD was < 0.6 (*r* = −0.024, *p* = 0.649, Additional file 1: Figure S3B), suggesting that at these levels, there was no influence on the HOI. Therefore, we decided to exclude a total of 32 plasma samples with OD > 0.6, and circumscribe our analyses to the 319 samples with OD <0.6. Among these 319 study samples, HOI exhibited a non-normal distribution, with a preponderance towards lower values (Additional file 1: Figure S4), and it was not influenced by age as there was no association with increasing age (*r* = 0.121, *p* = 0.524).

**Table 1:** Subject characteristics

Parameter	Cohort (*n* = 30)
Demographics	
Age, y	28 ± 9
Body mass index, kg/m^2^	23 ± 4
Sex, male/female	13/17
Blood laboratory tests	
Total cholesterol, mg/dL	169 ± 28
LDL-C, mg/dL	106 ± 26
HDL-C, mg/dL	51 ± 14
Triglycerides, mg/dL	68 ± 33

Values are mean ± SD

*LDL-c* low density lipoprotein –cholesterol, *HDL-c* high density lipoprotein-cholesterol

**Table 2:** PM_2.5_ mass, ozone levels and particle counts during exposure

Exposure measure	Filtered air	PM_2.5_	Ozone	PM_2.5_ + ozone
PM_2.5_, μg/m^3^	1.3 ± 8.0	148.5 ± 54.4	2.8 ± 11.7	132.4 ± 38.7
Ozone, μg/m^3^	20.8 ± 14.6	18.9 ± 11.9	221.1 ± 14.1	216.4 ± 11.9
Particle count (≥0.3 μm)	NA	652259 ± 460843	NA	616691 ± 539005
Particle count (≥2.0 μm)	NA	2987 ± 1918	NA	2792 ± 1963

Particle counts are average of 40 cycles, 3 min in duration, at a flow rate of 0.1 cfm. Counts are estimates upstream of the concentrator (detailed description in methods). Values are mean ± SD (*n* = 30)

*NA* Non assessed

### CAP but not ozone alters HDL antioxidant/anti-inflammatory capacity

**Fig. 2:**
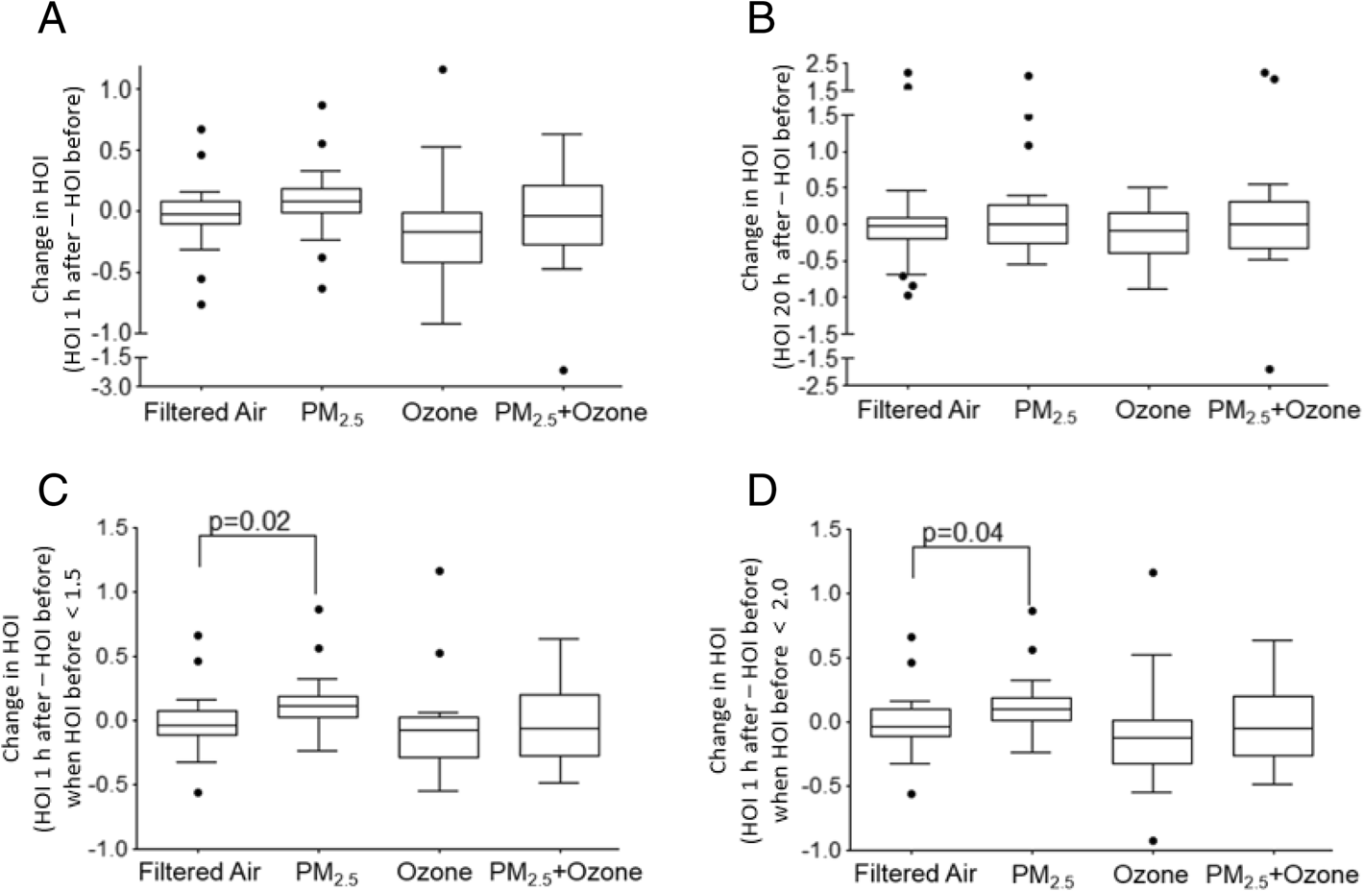
Effect of PM_2.5_ exposure on HDL antioxidant and anti-inflammatory function. **a** Change in HOI from before and 1 h after exposures. **b** Change in HOI from before and 20 h after exposures. **c** and **d** HOI from before and 1 h after exposures are shown in a subset where average pre HOI is <1.5 (**c**) and <2.0 (**d**). FA = filtered air

**Fig. 3:**
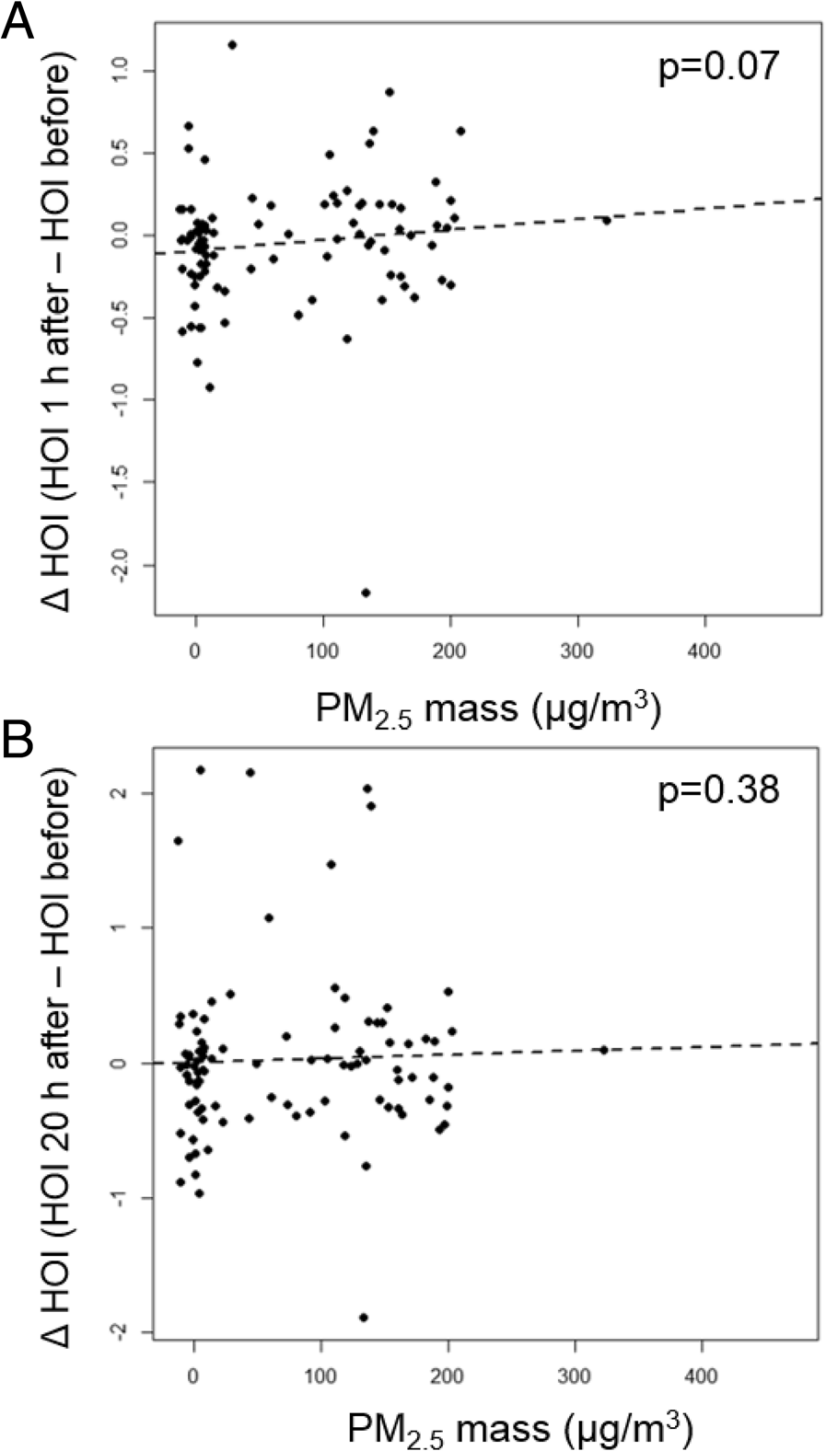
Association between PM_2.5_ mass concentration and ΔHOI. The PM_2.5_ mass concentration showed a trend to associate with ΔHOI 1 h after exposure (**a**) but not 20 h after exposure (**b**)

We evaluated the actual change in HOI (ΔHOI) as the difference in HOI from baseline, before exposure (Before) to 1 h after exposure (1 h after) or 20 h after exposure (20 h after). Although there were no significant differences in the mean HOI at each time point, among the various conditions (Table 3), there was a trend towards a bigger ΔHOI between PM_2.5_ and filtered air (*p* = 0.18) 1 h after exposure (Fig. 2a), that was absent 20 h after (Fig. 2b) or in the ozone or PM_2.5_ + ozone exposures, either 1 h after or 20 h after. We hypothesized that the PM_2.5_ -induced effects could be stronger when the baseline HOI was lower and subtle changes could be better detected. Indeed, we noticed that ΔHOI induced by PM_2.5_ was significantly larger, as compared with FA exposures, when the baseline HOI was < 1.5 (*p* = 0.019, Fig. 2c) or < 2.0 (*p* = 0.04, Fig. 2d) 1 h after exposure but not 20 h after. Interestingly, we observed that ΔHOI 1 h after exposure showed a positive trend with PM2.5 exposure mass concentration (*p* = 0.078, Fig. 3). We explored whether changes in HOI were associated with changes in PON-1 enzymatic activity, an important antioxidant enzyme associated with HDL, which we have found to be altered in hyperlipidemic mice exposed to concentrated ultrafine particles [23] or diesel exhaust [24]. However, we did not observe any significant differences in the enzymatic activity of PON-1 due to exposures at the different time points (Table 3). Altogether, these data suggests that PM_2.5_ exposure for only 2 h, induced effects on HDL anti-oxidant/anti-inflammatory function that were rather quick but resolved within 24 h after exposure, and not associated with alteration in PON-1 activity.

**Table 3:** Mean HOI and PON-1 activity

Exposure	Time	HOI	PON-1 activity
Filtered air	Before	1.13 ± 0.14	0.81 ± 0.07
	1 h after	1.05 ± 0.13	0.79 ± 0.08
	20 h after	1.12 ± 0.18	0.72 ± 0.07
PM_2.5_	Before	1.04 ± 0.15	0.77 ± 0.07
	1 h after	1.23 ± 0.17	0.81 ± 0.07
	20 h after	1.23 ± 0.20	0.76 ± 0.07
Ozone	Before	1.15 ± 0.16	0.76 ± 0.07
	1 h after	1.05 ± 0.14	0.81 ± 0.09
	20 h after	1.07 ± 0.18	0.78 ± 0.08
PM_2.5_ + ozone	Before	1.15 ± 016	0.78 ± 0.07
	1 h after	1.13 ± 0.14	0.83 ± 0.09
	20 h after	1.21 ± 0.18	0.82 ± 0.09

Values are mean ± SEM

PON-1 activity (μmol p-nitrophenol/min/ml plasma)

### Association between ΔHOI and systolic blood pressure

**Fig. 4:**
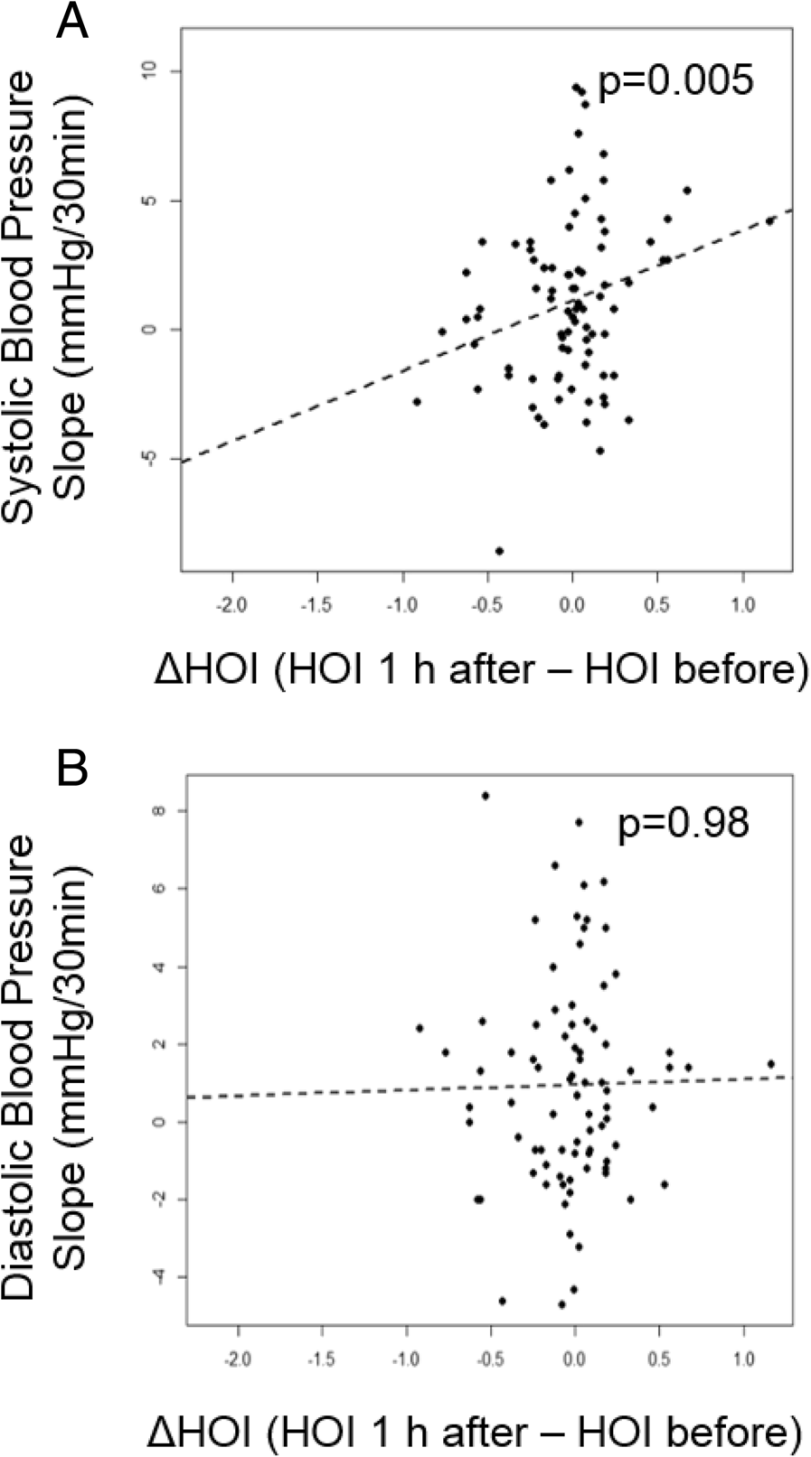
ΔHOI associates with systolic BP slope. Change in HOI 1 h after exposure was significantly associated with the systolic BP change observed during the 2 h exposure (**a**). ΔHOI after exposure did not correlate with diastolic BP slope (**b**)

PM_2.5_ containing exposures had been noted to increase SBP and DBP during the 2 h exposures [33]. Therefore, we explored whether there could be an association between effects on HOI and BP. Indeed, there was a strong positive association between ΔHOI with the systolic BP (SBP) slope (*p* = 0.005, Fig. 4a) but not diastolic BP (DBP) slope during exposure (Fig. 4b).

## Discussion

This is the first report describing significant changes in HDL functionality induced acutely by brief exposures to air pollutants in humans. We found that the HDL anti-oxidant/anti-inflammatory capacity significantly decreased from baseline among subjects with lower HOI pre-exposure values, following 2 h exposures to fine PM. HDL functional changes showed a positive correlation with the PM exposure mass, and strongly associated with elevations in systolic blood pressure during the exposures.

We used a fluorescent assay to evaluate the HDL anti-oxidant capacity via evaluating the ability of HDL to inhibit air-induced LDL oxidation, expressed as an index (HOI). The dysfunction in HDL anti-oxidant protection also indicates diminished HDL anti-inflammatory capacity, as judged by a monocyte chemotactic assay, with a strong correlation between both protective capacities [24, 29]. Therefore, the HOI serves as an instrumental measure of HDL anti-oxidant and anti-inflammatory capacities. This assay has shown to be quite sensitive and reproducible in previous studies [34, 35, 37] and similar assays have been employed to demonstrate impairment of HDL functionality in patients with CAD [26, 27, 29]. We hypothesized that assessment of HDL antioxidant protection could be affected by small degrees of hemolysis that might occur during processing of blood samples. We noted that HOI strongly correlated with the degree of hemolysis, assessed spectrophotometrically, when the OD was equal or above 0.6 at 410 nm, but not when the OD was less than 0.6, which is the reason why we excluded those samples from subsequent analyses. This data suggests this could be a helpful parameter to take into consideration when assessing HDL antioxidant protection with fluorescent assays.

PM exposure has been shown to induce systemic oxidative stress and atherosclerosis in various animal models, accompanied by changes in HDL functional status [38]. Dysfunctional HDL is emerging as a possible marker for PM-induced early CV effects, associated with lipid peroxidation in the blood [24]. In this context, it could exhibit higher sensitivity than other more traditional biomarkers of oxidative stress and inflammation. For instance, we reported that ApoE null mice exposed to ambient UFP for 5 weeks, exhibited proinflammatory HDL and enhanced atherosclerosis [13], at the same time when there were no significant changes in plasma levels of IL-6 and IL-8 (unpublished). Changes in HDL functional status could be valuable in PM-induced CV pathogenesis as they have been associated with the development and progression of atherosclerosis [25–27, 29, 39]. We hypothesized that PM_2.5_ and ozone exposures could lead to the development of dysfunctional HDL in humans, with higher HOIs, in a similar manner to diesel exhaust and ambient UFP in animal models of atherosclerosis [13, 23, 24]. As compared with FA exposures, HOI significantly increased after PM_2.5_ exposures, when baseline HOI was lower (HOI < 1.5 or < 2.0, Fig. 2), suggesting that PM-induced effects could be more easily detected when the HDL antioxidant capacity was stronger, likely indicative of a larger antioxidant reserve. It is also plausible that among subjects with HOI > 2.0, there could be pre-existing abnormalities in HDL function induced by any number of other health factors that could abrogate the ability for the modest impact of short-term PM_2.5_ exposure to be seen. Therefore, we believe it is indeed an important observation that HOI was adversely changed among individuals with more protective baseline HDL functionality prior to the exposures, which accounted for 89 % of all exposures. In addition, the change in HOI after exposure showed a positive trend to associate with PM_2.5_ exposure mass concentration (Fig. 3). This finding adds support to the interpretation of the data that PM_2.5_ can possibly affect HDL function. To our knowledge, this is the first report about the modifying effects of PM_2.5_ exposure on HDL functionality in humans. Interestingly, we have reported that 2-hour exposures to rural coarse PM (PM_2.5–10_) does not induce changes in HOI or HDL-mediated cholesterol efflux [37], which suggests the higher CV toxicity of urban PM_2.5_, as far as the ability to induce functional changes in plasma HDL.

Ozone co-exists with PM and their interaction could result in potentially additive harmful effects [40]. We tested the hypothesis that a co-exposure of PM_2.5_ and ozone would alter HDL function in a synergistic or additive manner but instead, neither ozone nor the combination of PM_2.5_ and ozone had any significant effect on the change in HOI compared to filtered air, in the entire study or subset of exposures with HOI <1.5 or <2.0. Interestingly, there was no effect of ozone alone on BP in the previous Clean Air report either, while the PM_2.5_ exposures caused a significant linear increase in DBP [33]. One possibility is that CV effects due to air pollution are much stronger with the PM components than with ozone, if at all present. Indeed, two recent meta-analyses have shown that the incidence of myocardial infarctions and heart failure hospitalizations were associated with PM_2.5_ and PM_10_, but not with ozone [41, 42]. On the other hand, some controlled exposure studies have shown that ozone potentiates the effects of PM_2.5_ on cardiovascular responses in humans, mice and rats. Combined particle and ozone exposure decreased heart rate variability in asthmatic adults [43] while ozone potentiated the effect of CAPs on diastolic BP (increased) in healthy young adults [44]. Studies on rats have shown that ozone co-exposure potentiates the effect of PM_2.5_ on heart rate variability [45–47]. The fact that the addition of ozone to fine PM blunted PM-induced effects on HOI suggests that ozone co-exposure or other changes in the aerosol chemical composition could have resulted in attenuation of fine PM toxicity by mechanisms not well understood [46–49]. However, while the concentrations of sulfur dioxide and nitric oxide were lower in the combined exposures (Table 4), it is unlikely they could have affected particle toxicity since sulfur dioxide levels were too low and differences were very small. The concentrations of nitric oxide were lower in the combined exposures due to its reaction with ozone to form nitrogen dioxide, which was present at higher concentrations in the combined exposures, but without significant differences in the concentrations of nitrogen oxides (nitric oxide + nitrogen dioxide). In addition, there were no differences in PM chemical composition, including the concentrations of nitrates, sulphates, ammonium or metals (Table 4) or any significant associations, after Bonferroni correction for multiple comparisons, between those concentrations present in all exposures and changes in HOI (Additional file 1: Table S1). It is possible to consider that the addition of ozone could have modified particle toxicity although there was only a few seconds of reaction time for a potential interaction between ozone and particles delivered to the subjects. Another possibility is that the addition of ozone could have induced alterations in the breathing pattern, by modulation of the pulmonary autonomic response, resulting in decreased tidal volumes and decreased airway particle deposition. This would be consistent with our previous report where some individuals exposed to PM_2.5_ + ozone exhibited shallow breathing and reduced IL-6 response in the blood [48]. However, we did not observe any associations between changes in HOI and respiratory frequency, tidal volume or minute ventilation.

**Table 4:** Chemical composition of PM_2.5_ and PM_2.5_ + ozone aerosols

Component	PM_2.5_	PM_2.5_ + ozone	*p* value
Ammonium (μg/m^3^)	4.7 ± 3.2	4.6 ± 4.8	0.919
Nitrate (μg/m^3^)	6.8 ± 6.2	5.7 ± 6.7	0.533
Sulphate (μg/m^3^)	11.1 ± 10.5	10.0 ± 10.9	0.693
Organic carbon (μg/m^3^)	28.0 ± 15.2	31.3 ± 16.5	0.425
Elemental carbon (μg/m^3^)	4.7 ± 3.1	4.5 ± 2.6	0.824
Total carbon (μg/m^3^)	32.7 ± 17.2	35.9 ± 17.4	0.488
Sulphur dioxide (μg/m^3^)	7.6 ± 13.6	1.9 ± 2.0^*^	0.03
Ozone (μg/m^3^)	18.9 ± 11.9	216.4 ± 11.9^*^	1.25 E-53
Nitric oxide (μg/m^3^)	23.9 ± 31.4	4.4 ± 3.5^*^	0.001
Nitrogen dioxide (μg/m^3^)	31.2 ± 19.7	48.2 ± 35.6^*^	0.026
Nitrogen oxide (μg/m^3^)	67.7 ± 53.1	54.7 ± 39.3	0.18
Total mass (μg/m^3^)	161.8 ± 59.7	157.7 ± 82.8	0.829

Nitrogen oxide = Nitric oxide + Nitrogen dioxide. Values are mean ± S.D. *p<0.05

We have previously reported that an increase in HOI, following exposure to diesel exhaust or ambient UFP in hyperlipidemic mice was associated with a concomitant decrease in PON-1 enzyme activity [23, 24]. However, we did not observe any effect of pollutants on PON-1 activity in the current study. We have reported that ApoE null mice exposed to diesel exhaust for 2 weeks exhibited a decrease in PON-1 activity that returned to baseline after 1 week of filtered air, even though the HOI remained elevated, indicating that dysfunctional HDL could persist as such in the presence of normal PON-1 enzymatic activity [24]. This indicates alternate mechanisms for the loss of HDL anti-oxidant/anti-inflammatory function. It is possible that PM exposures could have affected apoA1, which can exert antioxidant effects by binding oxidized lipids, or other antioxidant enzymes associated with HDL, such as platelet-activating factor- acetylhydrolase (PAF-AH) or lecithin:cholesterol acyltransferase (LCAT) [50]. LCAT activity is an important factor in HDL reverse cholesterol transport (RCT) function, which was not assessed in this study.

We also observed that the changes in HOI significantly correlated with the slope of SBP during exposures (Fig. 4). It has been reported that coarse PM exposure mass correlates with increases in SBP and heart rate [51], at the same time that arterial stiffness, which can be quantified by the augmentation index (ratio of augmented pressure to the pulse pressure), positively correlates with SBP and dyslipidemia [52]. It is possible that the positive association between SBP and HDL function in our study could be due to increased PM-induced arterial vasoconstriction. Mice fed an atherogenic diet develop proinflammatory HDL, which can induce vasoconstriction and impaired acetylcholine-mediated relaxation in mouse aortic rings [53]. In addition, HDL from CAD patients has been shown to activate the LOX-1 receptor on endothelial cells and thus to inhibit e-NOS dependent NO production [54]. This is the first report on a positive association between changes in HDL function and SBP, which suggests the possibility that defective HDL could promote vasomotor dysfunction. This could lead to altered hemodynamics and blood pressure measures, which will need to be validated in future studies.

HDL dysfunction is known to occur rapidly and to be reversible [28, 30]. We observed the effects of PM 1 h after exposure but not 20 h after. The 20-hour post-exposure values may not be linked to PM_2.5_ exposure due to diet, food and lifestyle impacts during the following day that we could not control for, beyond the fact that the changes may have resolved. The reversible effects of PM on HDL function could be due to the swift onset of oxidative stress rather than systemic inflammation, since there were no significant changes in blood biomarkers of inflammation in the previous Clean Air report [33].

One study limitation is that we did not evaluate other HDL protective facets such as reverse cholesterol transport. Our animals studies have shown that 2 to 10-week exposures to air pollutants result in marked effects on HDL antioxidant/antiinflammatory capacity that last for a few days, but without affecting HDL-mediated cholesterol efflux [24]. In the current study, HDL functional changes were transient and of a short duration, which will make it even less likely they would have involved effects on cholesterol efflux. However, identifying HDL changes after only 2-hour exposures to concentrated PM_2.5_ is quite important, even if those were of smaller magnitude and shorter duration than those observed in experimental animals since this suggests that brief exposures to fine PM have the potential to induce harmful vascular proinflammatory effects.

The PM_2.5_ levels used in this study are occupationally relevant in firefighters, locomotive workers, garage workers and miners [55]. Although the particle concentrations near highways are significantly lower in the US, these levels can be observed for short durations of time in many cities [33], particularly outside the US. The ozone exposure was at 110 ppb or 0.11 ppm (221 μg/m^3^) which is the maximum allowable ozone concentration in industrial working areas [56]. We believe that longer-term PM exposures, over several days, are much more likely to be capable of inducing larger and more persistent adverse effects on HDL function. Indeed, other studies show pro-inflammatory or endothelial dysfunction effects of PM_2.5_ exposures during a few days [57], while the effects of 2-hour controlled exposures are more modest and are more likely due to autonomic imbalance [33].

## Conclusions

In conclusion, we have found that brief PM_2.5_ exposures induce acute effects on HDL anti-oxidant and anti-inflammatory capacity, which strongly associated with changes in systolic blood pressure during the exposures. These findings may be important in identifying novel biomarkers for air pollutant-mediated cardiovascular toxicity and explaining potential mechanisms of how PM_2.5_ exposures induce cardiovascular events.

## Methods

### Study participants, study design and exposures

The current study used stored plasma samples collected from the Toronto component of controlled human exposure studies performed as part of the Clean Air research center project in Michigan and Toronto. The study was approved by the human research ethics committee of the University of Toronto. The inclusion and exclusion criteria for study participants, study design and exposure protocol have been described previously [33]. Briefly, subjects were healthy 18- to 50-year old non-smokers without any risk for cardiovascular disease and were not taking any medications during the exposure period. Subjects were pre-screened using blood biochemistry and were excluded when values for fasting total cholesterol and glucose were >240 mg/dl or >126 mg/dl respectively. A total of 30 subjects with 13 males and 17 females participated in the study. The exposures were conducted at the Gage controlled human inhalation facility, University of Toronto. The study design involved randomized blinded exposures to four different conditions: filtered air, concentrated fine PM (PM_2.5_), ozone and concentrated fine PM plus ozone (PM_2.5_ + ozone) for 2 h. PM_2.5_ and ozone exposures were performed and monitored as described in the parent study [33]. Briefly, ambient PM_2.5_ was concentrated to a target level of 150 μg/m^3^ with a 2-stage Harvard virtual impactor system. PM_2.5_ levels were monitored during exposures by a tapered element oscillating microbalance (Rupprecht & Patashnick Co., Inc., model 1400a. Particle counts were obtained using a Met One laser particle counter (Pacific Scientific Co., model 237B). The Met One sampled the outside air 1.8 m below the concentrator inlet. Ambient particle counts were measured in 6 size channels (0.3, 0.5, 0.7, 1.0, 2.0 and 5.0 μm) at a flow rate of 0.1 cubic feet per minute (cfm). The Met One was run for 40 cycles of 3 min and the average of the 40 3-min cycles was reported for particles 0.3 μm and larger and 2.0 μm and larger. The short 3-min sample periods were required, as longer periods would have saturated the count channels. Exposure particle counts were not measured in the concentrated PM_2.5_ air stream due to instrument limitations (negative pressure). During exposures with PM_2.5_, HEPA-filtered dilution air (DA) was added to the ambient air upstream of the concentrator to maintain exposure PM_2.5_ at the target concentration. Particle counts were estimated upstream of the PM_2.5_ concentrator using the concentrator inlet flow rate of 1100 litres/minute and the average flow rate of added DA, measured continuously during the exposure, as follows: ambient particle counts x (1100 - DA)/1100, as described previously [58]. During filtered air and ozone exposures an in-line high efficiency particulate air filter was inserted downstream of the concentrator, thus particle counts were zero (testing confirmed this). Ozone (240 μg/m^3^ or 120 ppb) was produced by an arc generator and added to the particle airflow. Ozone was monitored continuously by using a Dasibi (model 1008RS) photometric analyzer. A PM_2.5_ filter sample was collected immediately upstream of the chamber on a 47-mm Gelman Teflon filter with a 2 μm pore size at an air flow of 8 L/min. The sample was analyzed gravimetrically for total mass on conditioned filters using a climate-controlled clean room under stable conditions of 37 ± 5 % relative humidity and 21 ± °C [58]. Ambient gases were measured using continuous reading gas analyzers including: SO_2_ (Monitor Labs, model 8850); nitrogen oxides (NO, NO_2_, Monitor Labs model 8840); and CO (Thermo Electron Instruments, model 48). Readings were logged every 15 s and averaged over the 2-hour exposure. Gases were measured after PM concentrating before delivery to the subjects. CO, SO_2_, NO, NO_2_ were measured directly. Nitrogen oxides (NO_X_) were calculated as NO + NO_2_.

Each participant received all 4 exposures at least two weeks apart. Blood pressure (BP) was determined every 30 min during the 2 h exposures. Blood was collected before exposure (before), 1 h after exposure (1 h after) and the following day, approximately 20 h after exposure (20 h after). Subjects were exposed to filtered air, concentrated PM_2.5_, ozone, or PM_2.5_ + ozone, for 2 h, in a blindly randomized sequence with a cross-over design (Additional file 1: Figure S1). 28 subjects received all 4 exposures (336 blood samples), 2 subjects received 2 exposures each (12 blood samples) and one subject received an extra 5^th^ exposure (3 blood samples), which yielded a total of 351 stored plasma samples. Subjects displayed a normal range of lipid and glucose levels representing a healthy cohort (Table 1). The average concentrations of PM_2.5_ and ozone in the PM_2.5_ only and ozone only containing exposures were 149 ± 54 μg/m^3^ and 221 ± 14 μg/m^3^ (111 ± 7 ppb), respectively. The particle and ozone concentrations were 132 ± 39 μg/m^3^ and 216 ± 12 μg/m^3^ (109 ppb), respectively in the co-exposures (Table 2).

### HDL anti-oxidant and anti-inflammatory capacity

HDL anti-oxidant capacity was determined as the ability of HDL to inhibit LDL-induced oxidation of dihydrodichlorofluorescein (DCFH) into the fluorescent dichlorofluorescein (DCF). The assay was performed as previously described [24, 50]. Briefly, HDL cholesterol (HDL-c) was isolated from plasma using the dextran-sulfate precipitation method and the HDL-c concentration was determined using the Infinity cholesterol reagent and a cholesterol standard (Thermo Scientific, Middletown, VA). This method of HDL separation shows the same effect on anti-oxidant capacity as HDL obtained by ultracentrifugation [50]. The dichlorofluorescein diacetate (DCF-DA) (Sigma-Aldrich, St. Louis, MO) was incubated with NaOH to induce alkaline hydrolysis of DCF-DA to DCFH. The mixture was then neutralized with 10X phosphate-buffered saline (PBS). Each HDL sample was mixed with an equal concentration LDL cholesterol (50 μg /ml) and made up to a total volume of 100 μl with Tris–HCl buffer (pH 7.4). The mixture was then added to a black well flat bottom microtiter plate in triplicate and the plate was incubated at 37 °C for 1 h. Next, 20 μl of the DCFH reagent was added to each well and the plate was incubated at 37 °C for 3 h. DCF fluorescence intensity was determined using a plate reader (SynergyMx, BioTek, Vermont) at an excitation wavelength of 485 nm and emission wavelength of 530 nm. HDL anti-oxidant capacity is expressed as the HDL oxidant index (HOI), determined by the ratio of DCF fluorescence in the presence and absence of HDL cholesterol. An index < 1.0 denotes protective anti-oxidant HDL whereas an index > 1.0 indicates pro-oxidant HDL. Our assay has shown to be robust and reproducible with inter- and intra-assay coefficient of variations of 7.30 % and 6.89 % respectively, as previously reported [35]. This assay has also shown a high inter-user correlation, *r* = 0.74, *p* = 0.0003 (Additional file 1: Figure S2) [34].

### Paraoxonase assay

Paraoxonase-1 (PON-1) is an HDL-associated anti-oxidant enzyme and its activity was determined as its ability to hydrolyze paraoxon substrate (diethyl-*p*-nitrophenyl phosphate) to *p*-nitrophenol, a characteristic of only the PON-1 isoform [59]. The assay method has been described before [24]. Briefly, 5 μl of plasma was incubated with 2.4 mM of paraoxon substrate in 0.1 M Tris–HCl buffer (pH 8.5) buffer containing 2 mM CaCl_2_ and 2.0 M NaCl at room temperature. The kinetics of *p*-nitrophenol formation was determined by recording absorbance at 405 nm every 15 s for 4 min. PON-1 enzyme activity was expressed as μmol *p*-nitrophenol formed per minute for every 1 mL of plasma.

### Statistical analysis

To examine the change in HOI (ΔHOI) induced by exposures, the before (baseline) HOI was subtracted from the 1 h after or 20 h after HOI in all exposures. Differences between the ΔHOI for each exposure were tested using the Wilcoxon non-parametric test. Each subject received all four exposures. A linear mixed effects model, with subjects as the random effects to control for within subject correlation of repeated measures, was used to determine the association among ΔHOI, PM_2.5_ mass concentration, systolic and diastolic blood pressure with all exposures. All tests were two-sided and a p-value less than 0.05 was considered statistically significant. Data were analyzed using SAS version 9.1 (SAS Institute).

## Additional file

Additional file 1: Figure S1. Exposure Design**.** CAP, concentrated ambient particles; BP, blood pressure. Figure adapted and modified from [33]. **Figure S2.** HDL oxidant index is stable and reproducible. The inter-user correlation is shown, *r* = 0.74, *p* = 0.0003. **Figure S3.** Effect of Hemolysison HOI. Hemolysis was determined spectrophotometry at 410 nm. Increased OD readings correlated with increased HOI when all samples were plotted (A). There was a threshold effect assamples with OD >0.6 exhibited the association but samples with OD <0.6 did not (B), suggesting that hemolyses did not influence HOI at these levels. Therefore, we excluded samples with OD >0.6 from subsequent analyses. Hemolysis did not correlate with PON-1 activity when all samples were plotted (C). **Figure S4.** HOI distribution. The distribution of HOI across the entire population of study subjects was non-normal (A). Logarithmic transformation, however, resulted in normally distributed data (B). **Table S1.** Concentration of metals in PM_2.5_ and PM_2.5_ + Ozone exposures. (PDF 468 kb)

## Abbreviations

ApoE: apolipoprotein E
CAP: concentrated ambient particles
CV: cardiovascular
HDL: high density lipoprotein
HOI: HDL oxidant index
LDL-r: low density lipoprotein receptor
PM: particulate matter
UFP: ultrafine particles

## References

[1] Brook RD, Rajagopalan S, Pope CA, Brook JR, Bhatnagar A, Diez-Roux AV, Holguin F, Hong Y, Luepker RV, Mittleman MA (2010). Particulate matter air pollution and cardiovascular disease: An update to the scientific statement from the American Heart Association. Circulation.

[2] Pope CA, Burnett RT, Thurston GD, Thun MJ, Calle EE, Krewski D, Godleski JJ (2004). Cardiovascular mortality and long-term exposure to particulate air pollution: epidemiological evidence of general pathophysiological pathways of disease. Circulation.

[3] Kunzli N, Jerrett M, Mack WJ, Beckerman B, LaBree L, Gilliland F, Thomas D, Peters J, Hodis HN (2005). Ambient air pollution and atherosclerosis in Los Angeles. Environ Health Perspect.

[4] Hoffmann B, Moebus S, Mohlenkamp S, Stang A, Lehmann N, Dragano N, Schmermund A, Memmesheimer M, Mann K, Erbel R (2007). Residential exposure to traffic is associated with coronary atherosclerosis. Circulation.

[5] Diez Roux AV, Auchincloss AH, Franklin TG, Raghunathan T, Barr RG, Kaufman J, Astor B, Keeler J (2008). Long-term exposure to ambient particulate matter and prevalence of subclinical atherosclerosis in the Multi-Ethnic Study of Atherosclerosis. Am J Epidemiol.

[6] Allen RW, Criqui MH, Diez Roux AV, Allison M, Shea S, Detrano R, Sheppard L, Wong ND, Stukovsky KH, Kaufman JD (2009). Fine particulate matter air pollution, proximity to traffic, and aortic atherosclerosis. Epidemiology.

[7] Kunzli N, Jerrett M, Garcia-Esteban R, Basagana X, Beckermann B, Gilliland F, Medina M, Peters J, Hodis HN, Mack WJ (2010). Ambient air pollution and the progression of atherosclerosis in adults. PLoS One.

[8] Adar SD, Sheppard L, Vedal S, Polak JF, Sampson PD, Diez Roux AV, Budoff M, Jacobs DR, Barr RG, Watson K, Kaufman JD (2013). Fine particulate air pollution and the progression of carotid intima-medial thickness: a prospective cohort study from the multi-ethnic study of atherosclerosis and air pollution. PLoS Med.

[9] Chen T, Jia G, Wei Y, Li J (2013). Beijing ambient particle exposure accelerates atherosclerosis in ApoE knockout mice. Toxicol Lett.

[10] Wan Q, Cui X, Shao J, Zhou F, Jia Y, Sun X, Zhao X, Chen Y, Diao J, Zhang L (2014). Beijing ambient particle exposure accelerates atherosclerosis in ApoE knockout mice by upregulating visfatin expression. Cell Stress Chaperones.

[11] Chen LC, Nadziejko C (2005). Effects of subchronic exposures to concentrated ambient particles (CAPs) in mice. V. CAPs exacerbate aortic plaque development in hyperlipidemic mice. Inhal Toxicol.

[12] Sun Q, Wang A, Jin X, Natanzon A, Duquaine D, Brook RD, Aguinaldo JG, Fayad ZA, Fuster V, Lippmann M (2005). Long-term air pollution exposure and acceleration of atherosclerosis and vascular inflammation in an animal model. JAMA.

[13] Araujo JA, Barajas B, Kleinman M, Wang X, Bennett BJ, Gong KW, Navab M, Harkema J, Sioutas C, Lusis AJ, Nel AE (2008). Ambient particulate pollutants in the ultrafine range promote early atherosclerosis and systemic oxidative stress. Circ Res.

[14] Quan C, Sun Q, Lippmann M, Chen LC (2010). Comparative effects of inhaled diesel exhaust and ambient fine particles on inflammation, atherosclerosis, and vascular dysfunction. Inhal Toxicol.

[15] Miller MR, McLean SG, Duffin R, Lawal AO, Araujo JA, Shaw CA, Mills NL, Donaldson K, Newby DE, Hadoke PW (2013). Diesel exhaust particulate increases the size and complexity of lesions in atherosclerotic mice. Part Fibre Toxicol.

[16] Niwa Y, Hiura Y, Murayama T, Yokode M, Iwai N (2007). Nano-sized carbon black exposure exacerbates atherosclerosis in LDL-receptor knockout mice. Circ J.

[17] Campen MJ, Lund AK, Doyle-Eisele ML, McDonald JD, Knuckles TL, Rohr AC, Knipping EM, Mauderly JL (2010). A comparison of vascular effects from complex and individual air pollutants indicates a role for monoxide gases and volatile hydrocarbons. Environ Health Perspect.

[18] Campen MJ, Lund AK, Knuckles TL, Conklin DJ, Bishop B, Young D, Seilkop S, Seagrave J, Reed MD, McDonald JD (2010). Inhaled diesel emissions alter atherosclerotic plaque composition in ApoE(−/−) mice. Toxicol Appl Pharmacol.

[19] Chuang GC, Yang Z, Westbrook DG, Pompilius M, Ballinger CA, White CR, Krzywanski DM, Postlethwait EM, Ballinger SW (2009). Pulmonary ozone exposure induces vascular dysfunction, mitochondrial damage, and atherogenesis. Am J Physiol Lung Cell Mol Physiol.

[20] Breton CV, Wang X, Mack WJ, Berhane K, Lopez M, Islam TS, Feng M, Lurmann F, McConnell R, Hodis HN (2012). Childhood air pollutant exposure and carotid artery intima-media thickness in young adults. Circulation.

[21] Navab M, Reddy ST, Van Lenten BJ, Fogelman AM (2011). HDL and cardiovascular disease: atherogenic and atheroprotective mechanisms. Nat Rev Cardiol.

[22] Yeatts K, Svendsen E, Creason J, Alexis N, Herbst M, Scott J, Kupper L, Williams R, Neas L, Cascio W (2007). Coarse particulate matter (PM2.5-10) affects heart rate variability, blood lipids, and circulating eosinophils in adults with asthma. Environ Health Perspect.

[23] Li R, Navab M, Pakbin P, Ning Z, Navab K, Hough G, Morgan TE, Finch CE, Araujo JA, Fogelman AM (2013). Ambient ultrafine particles alter lipid metabolism and HDL anti-oxidant capacity in LDLR-null mice. J Lipid Res.

[24] Yin F, Lawal A, Ricks J, Fox JR, Larson T, Navab M, Fogelman AM, Rosenfeld ME, Araujo JA (2013). Diesel exhaust induces systemic lipid peroxidation and development of dysfunctional pro-oxidant and pro-inflammatory high-density lipoprotein. Arterioscler Thromb Vasc Biol.

[25] Khera AV, Cuchel M, de la Llera-Moya M, Rodrigues A, Burke MF, Jafri K, French BC, Phillips JA, Mucksavage ML, Wilensky RL (2011). Cholesterol efflux capacity, high-density lipoprotein function, and atherosclerosis. N Engl J Med.

[26] Patel PJ, Khera AV, Jafri K, Wilensky RL, Rader DJ (2011). The anti-oxidative capacity of high-density lipoprotein is reduced in acute coronary syndrome but not in stable coronary artery disease. J Am Coll Cardiol.

[27] Patel PJ, Khera AV, Wilensky RL, Rader DJ (2013). Anti-oxidative and cholesterol efflux capacities of high-density lipoprotein are reduced in ischaemic cardiomyopathy. Eur J Heart Fail.

[28] Van Lenten BJ, Hama SY, de Beer FC, Stafforini DM, McIntyre TM, Prescott SM, La Du BN, Fogelman AM, Navab M (1995). Anti-inflammatory HDL becomes pro-inflammatory during the acute phase response. Loss of protective effect of HDL against LDL oxidation in aortic wall cell cocultures. J Clin Invest.

[29] Navab M, Hama SY, Hough GP, Subbanagounder G, Reddy ST, Fogelman AM (2001). A cell-free assay for detecting HDL that is dysfunctional in preventing the formation of or inactivating oxidized phospholipids. J Lipid Res.

[30] Van Lenten BJ, Wagner AC, Nayak DP, Hama S, Navab M, Fogelman AM (2001). High-density lipoprotein loses its anti-inflammatory properties during acute influenza a infection. Circulation.

[31] Charles-Schoeman C, Lee YY, Grijalva V, Amjadi S, FitzGerald J, Ranganath VK, Taylor M, McMahon M, Paulus HE, Reddy ST (2012). Cholesterol efflux by high density lipoproteins is impaired in patients with active rheumatoid arthritis. Ann Rheum Dis.

[32] McMahon M, Grossman J, FitzGerald J, Dahlin-Lee E, Wallace DJ, Thong BY, Badsha H, Kalunian K, Charles C, Navab M (2006). Proinflammatory high-density lipoprotein as a biomarker for atherosclerosis in patients with systemic lupus erythematosus and rheumatoid arthritis. Arthritis Rheum.

[33] Brook RD, Urch B, Dvonch JT, Bard RL, Speck M, Keeler G, Morishita M, Marsik FJ, Kamal AS, Kaciroti N (2009). Insights into the mechanisms and mediators of the effects of air pollution exposure on blood pressure and vascular function in healthy humans. Hypertension.

[34] Breton CV, Yin F, Wang X, Avol E, Gilliland FD, Araujo JA (2014). HDL anti-oxidant function associates with LDL level in young adults. Atherosclerosis.

[35] Ramanathan G, Araujo JA, Gornbein J, Yin F, Middlekauff HR (2014). Cigarette smoking is associated with dose-dependent adverse effects on paraoxonase activity and fibrinogen in young women. Inhal Toxicol.

[36] Rubinstein DL, Ravikovich HM (1946). Absorption spectrum of haemoglobin in red cells. Nature.

[37] Maiseyeu A, Yang HY, Ramanathan G, Yin F, Bard RL, Morishita M, Dvonch JT, Wang L, Spino C, Mukherjee B (2014). No effect of acute exposure to coarse particulate matter air pollution in a rural location on high-density lipoprotein function. Inhal Toxicol.

[38] Araujo JA (2010). Particulate air pollution, systemic oxidative stress, inflammation, and atherosclerosis. Air Qual Atmos Health.

[39] Ansell BJ, Navab M, Hama S, Kamranpour N, Fonarow G, Hough G, Rahmani S, Mottahedeh R, Dave R, Reddy ST, Fogelman AM (2003). Inflammatory/antiinflammatory properties of high-density lipoprotein distinguish patients from control subjects better than high-density lipoprotein cholesterol levels and are favorably affected by simvastatin treatment. Circulation.

[40] Madden MC, Richards JH, Dailey LA, Hatch GE, Ghio AJ (2000). Effect of ozone on diesel exhaust particle toxicity in rat lung. Toxicol Appl Pharmacol.

[41] Mustafic H, Jabre P, Caussin C, Murad MH, Escolano S, Tafflet M, Perier MC, Marijon E, Vernerey D, Empana JP, Jouven X (2012). Main air pollutants and myocardial infarction: a systematic review and meta-analysis. JAMA.

[42] Shah AS, Langrish JP, Nair H, McAllister DA, Hunter AL, Donaldson K, Newby DE, Mills NL (2013). Global association of air pollution and heart failure: a systematic review and meta-analysis. Lancet.

[43] Power KL, Balmes J, Solomon C (2008). Controlled exposure to combined particles and ozone decreases heart rate variability. J Occup Environ Med.

[44] Fakhri AA, Ilic LM, Wellenius GA, Urch B, Silverman F, Gold DR, Mittleman MA (2009). Autonomic effects of controlled fine particulate exposure in young healthy adults: effect modification by ozone. Environ Health Perspect.

[45] Wang G, Jiang R, Zhao Z, Song W (2013). Effects of ozone and fine particulate matter (PM(2.5)) on rat system inflammation and cardiac function. Toxicol Lett.

[46] Kurhanewicz N, McIntosh-Kastrinsky R, Tong H, Walsh L, Farraj A, Hazari MS (2014). Ozone co-exposure modifies cardiac responses to fine and ultrafine ambient particulate matter in mice: concordance of electrocardiogram and mechanical responses. Part Fibre Toxicol.

[47] Wagner JG, Allen K, Yang HY, Nan B, Morishita M, Mukherjee B, Dvonch JT, Spino C, Fink GD, Rajagopalan S (2014). Cardiovascular depression in rats exposed to inhaled particulate matter and ozone: effects of diet-induced metabolic syndrome. Environ Health Perspect.

[48] Urch B, Speck M, Corey P, Wasserstein D, Manno M, Lukic KZ, Brook JR, Liu L, Coull B, Schwartz J (2010). Concentrated ambient fine particles and not ozone induce a systemic interleukin-6 response in humans. Inhal Toxicol.

[49] Sun L, Liu C, Xu X, Ying Z, Maiseyeu A, Wang A, Allen K, Lewandowski RP, Bramble LA, Morishita M (2013). Ambient fine particulate matter and ozone exposures induce inflammation in epicardial and perirenal adipose tissues in rats fed a high fructose diet. Part Fibre Toxicol.

[50] Yin F, Ramanathan G, Zhang M, Araujo JA (2013). Prooxidative effects of ambient pollutant chemicals are inhibited by HDL. J Biochem Mol Toxicol.

[51] Morishita M, Bard RL, Wang L, Das R, Dvonch JT, Spino C, Mukherjee B, Sun Q, Harkema JR, Rajagopalan S, Brook RD. The characteristics of coarse particulate matter air pollution associated with alterations in blood pressure and heart rate during controlled exposures. J Expo Sci Environ Epidemiol. 2015;25(2):153-9. 10.1038/jes.2014.62 PMC4462122 25227729

[52] Rosenbaum D, Giral P, Chapman J, Rached FH, Kahn JF, Bruckert E, Girerd X (2013). Radial augmentation index is a surrogate marker of atherosclerotic burden in a primary prevention cohort. Atherosclerosis.

[53] Watanabe J, Chou KJ, Liao JC, Miao Y, Meng HH, Ge H, Grijalva V, Hama S, Kozak K, Buga G (2007). Differential association of hemoglobin with proinflammatory high density lipoproteins in atherogenic/hyperlipidemic mice. A novel biomarker of atherosclerosis. J Biol Chem.

[54] Besler C, Heinrich K, Rohrer L, Doerries C, Riwanto M, Shih DM, Chroni A, Yonekawa K, Stein S, Schaefer N (2011). Mechanisms underlying adverse effects of HDL on eNOS-activating pathways in patients with coronary artery disease. J Clin Invest.

[55] National Toxicology P (2011). NTP 12th Report on Carcinogens. Rep Carcinog.

[56] Korczynski RE (2000). Occupational health concerns in the welding industry. Appl Occup Environ Hyg.

[57] O'Toole TE, Hellmann J, Wheat L, Haberzettl P, Lee J, Conklin DJ, Bhatnagar A, Pope CA (2010). Episodic exposure to fine particulate air pollution decreases circulating levels of endothelial progenitor cells. Circ Res.

[58] Urch B, Brook JR, Wasserstein D, Brook RD, Rajagopalan S, Corey P, Silverman F (2004). Relative contributions of PM2.5 chemical constituents to acute arterial vasoconstriction in humans. Inhal Toxicol.

[59] Aldridge WN (1953). Serum esterases. I. Two types of esterase (A and B) hydrolysing p-nitrophenyl acetate, propionate and butyrate, and a method for their determination. Biochem J.

