# Supplementary material for: Effects of urban fine particulate matter and ozone on HDL functionality
Source: Part Fibre Toxicol. 2016 May 24;13:26. doi: 10.1186/s12989-016-0139-3 (PMC4879751; doi:10.1186/s12989-016-0139-3)
Supplement: Additional file 1: Figure S1. — Exposure Design. CAP, concentrated ambient particles; BP, blood pressure. Figure adapted and modified from [33]. Figure S2. HDL oxidant index is stable and reproducible. The inter-user correlation is shown, r = 0.74, p = 0.0003. Figure S3. Effect of Hemolysison HOI. Hemolysis was determined spectrophotometry at 410 nm. Increased OD readings correlated with increased HOI when all samples were plotted (A). There was a threshold effect assamples with OD >0.6 exhibited the association but samples with OD <0.6 did not (B), suggesting that hemolyses did not influence HOI at these levels. Therefore, we excluded samples with OD >0.6 from subsequent analyses. Hemolysis did not correlate with PON-1 activity when all samples were plotted (C). Figure S4. HOI distribution. The distribution of HOI across the entire population of study subjects was non-normal (A). Logarithmic transformation, however, resulted in normally distributed data (B). Table S1. Concentration of metals in PM2.5 and PM2.5 + Ozone exposures. (PDF 468 kb) [file 12989_2016_139_MOESM1_ESM.pdf]

Supplementary figures

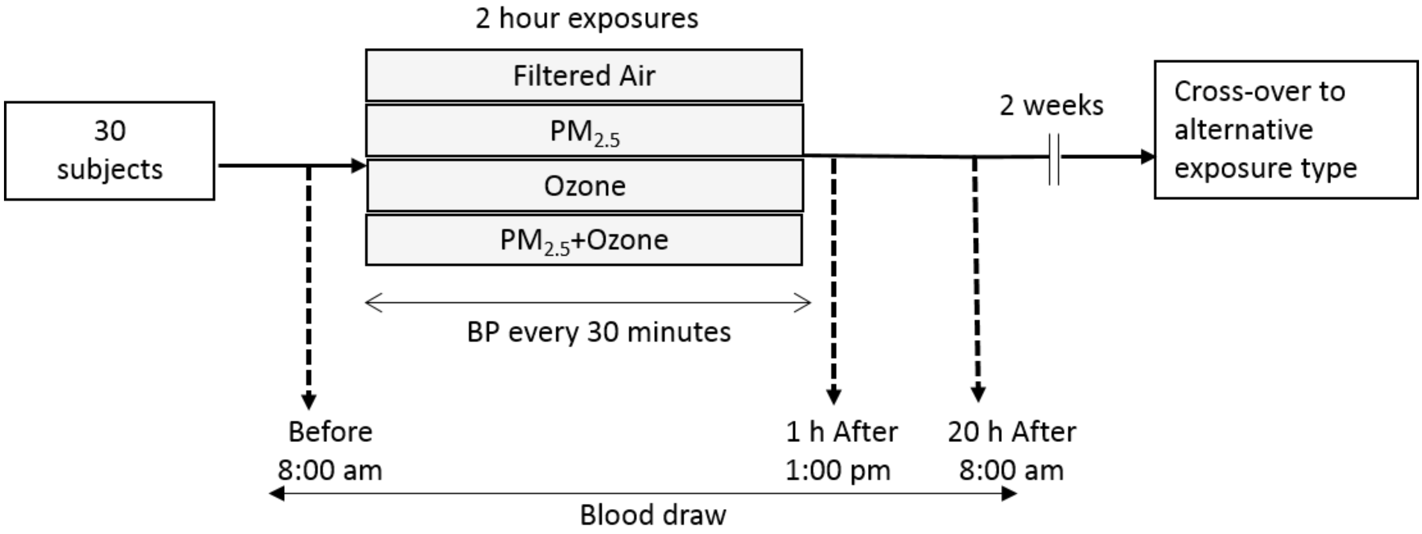

Figure S1. Exposure Design.

CAP, concentrated ambient particles; BP, blood pressure.

Figure adapted and modified from [33].

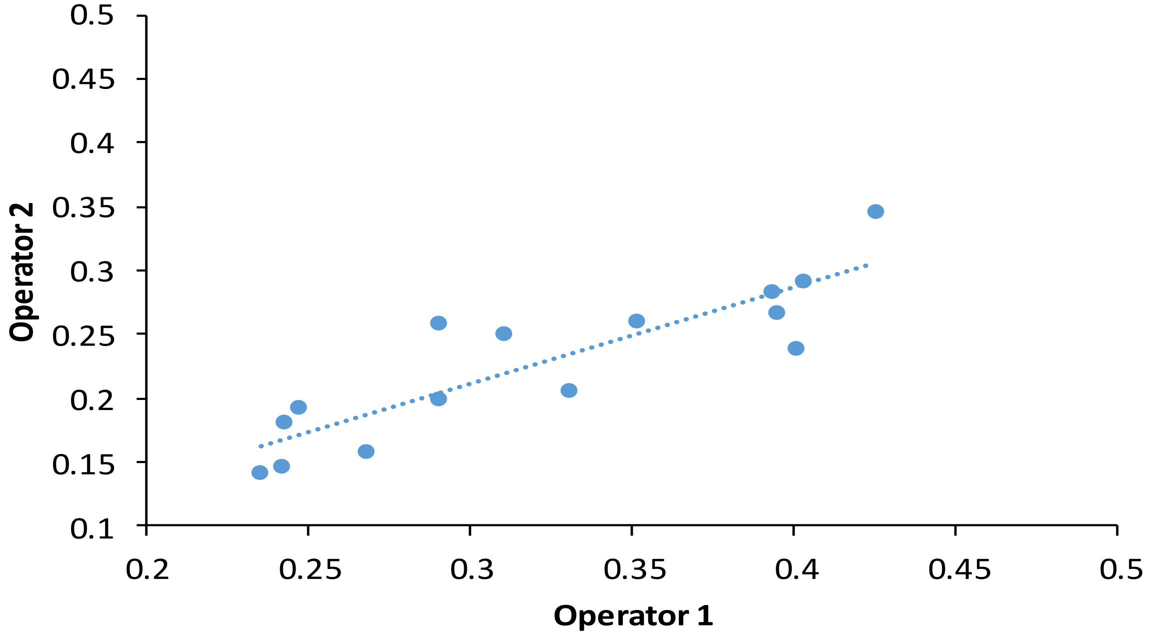

Figure S2. HDL oxidant index is stable and reproducible. The inter-user correlation is shown, r=0.74, p=0.0003.

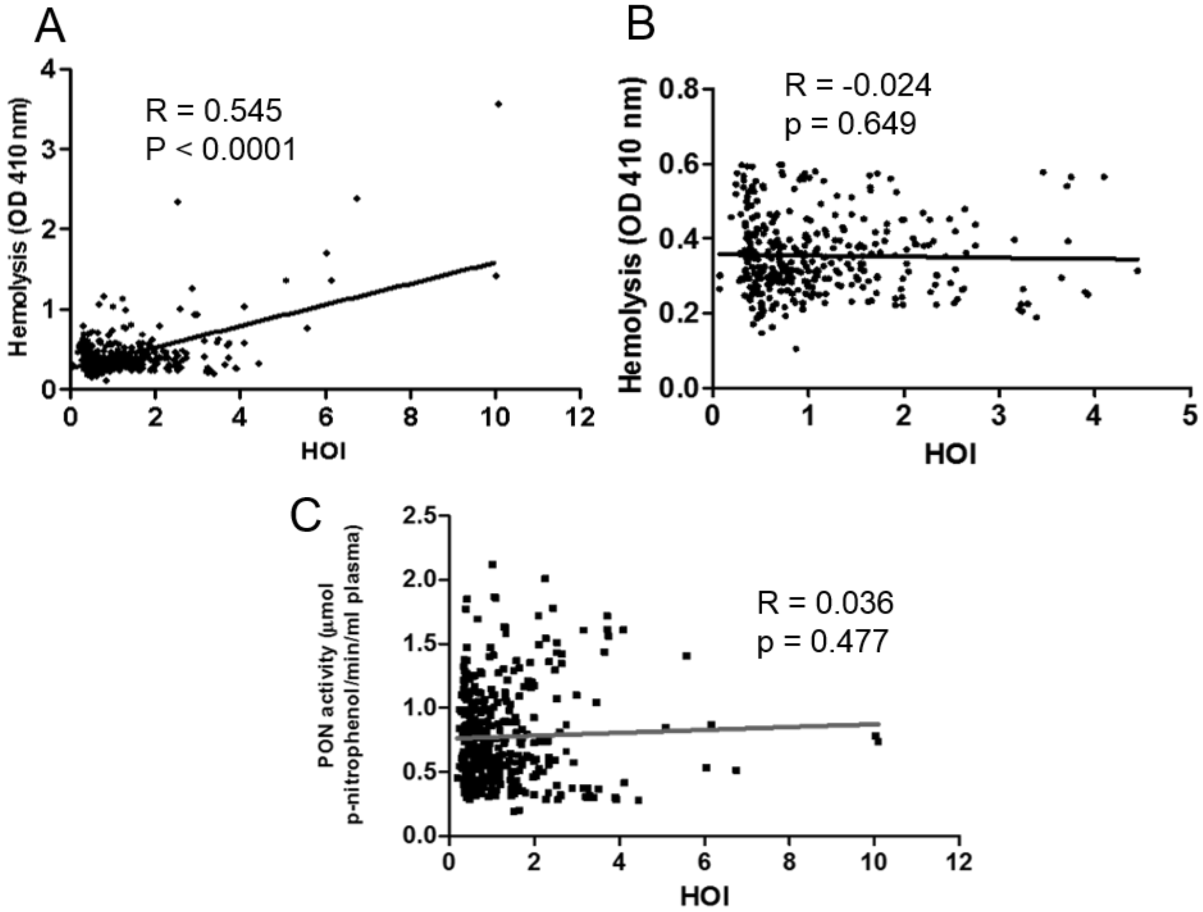

Figure S3. Effect of Hemolysis on HOI. Hemolysis was determined spectrophotometry at 410 nm. Increased OD readings correlated with increased HOI when all samples were plotted (A). There was a threshold effect as samples with OD > 0.6 exhibited the association but samples with OD < 0.6 did not (B), suggesting that hemolyses did not influence HOI at these levels. Therefore, we excluded samples with OD > 0.6 from subsequent analyses. Hemolysis did not correlate with PON-1 activity when all samples were plotted (C).

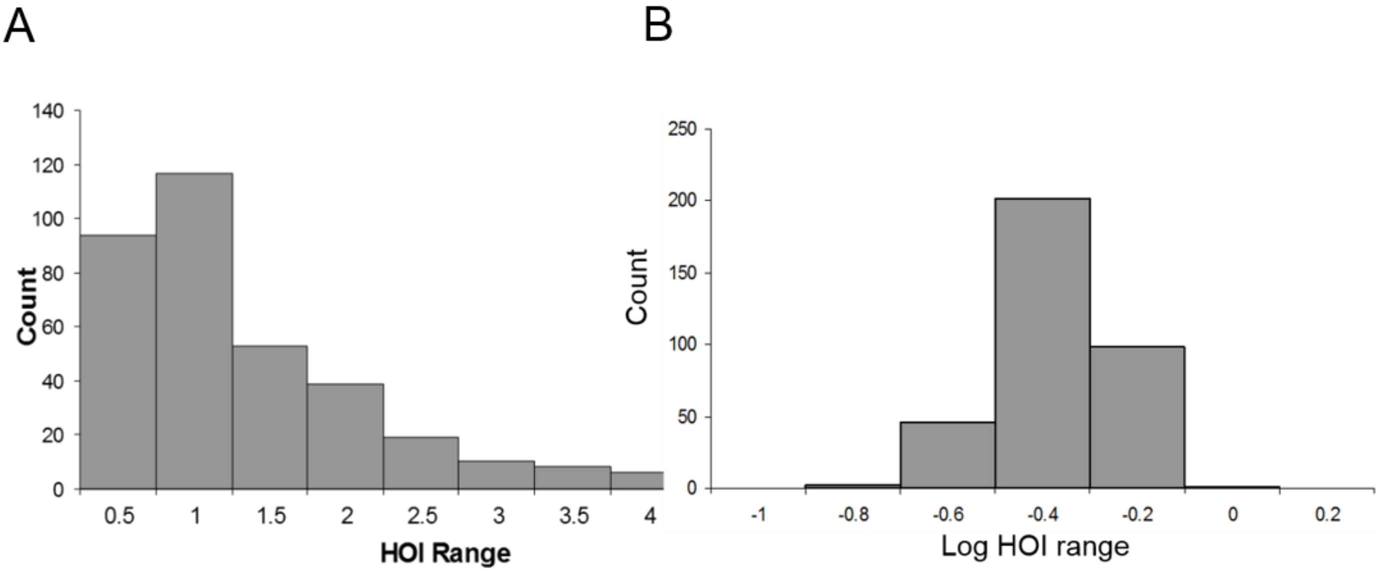

Figure S4. HOI distribution. The distribution of HOI across the entire population of study subjects was non-normal (A). Logarithmic transformation, however, resulted in normally distributed data (B).

Table S1. Concentration of metals in $PM_{2.5}$ and $PM_{2.5}$ +Ozone exposures.

Metal	PM2.5	PM2.5+Ozone	P value	Bonferroni p value
Chromium	20.7±39.1	22.4±34.8	0.853	>0.99
Magnesium	97.5±52.6	68.8±38.4	0.019	0.492
Iron	2683.5±1172.4	2004.9±1020.2	0.020	0.521
Cobalt	0.606±0.4	0.519±0.49	0.476	>0.99
Nickel	5.8±8.4	7.3±15.3	0.652	>0.99
Copper	92.6±87.3	77.4±84.9	0.497	>0.99
Zinc	413.0±356.8	269.8±281.5	0.090	>0.99
Potassium	824.5±459.8	738.5±351.5	0.419	>0.99
Arsenic	10.7±6.8	8.3±6.7	0.163	>0.99
Selenium	19.5±18.5	12.4±12.2	0.086	>0.99
Magnesium	986.1±514.4	884.3±561.0	0.467	>0.99
Rubidium	3.05±1.9	2.1±1.3	0.050	>0.99
Aluminium	1466.2±903.6	1411.3±819.2	0.806	>0.99
Strontium	15.8±8.7	14.3±8.4	0.509	>0.99
Molybdenum	4.4±1.7	3.7±1.8	0.137	>0.99
Cadmium	1.8±1.3	1.7±1.5	0.963	>0.99
Tin	18.3±24.4	10.5±16.1	0.149	>0.99
Antimony	18.7±11.8	15.3±10.6	0.255	>0.99
Lanthanum	1.09±0.7	1.0±0.8	0.959	>0.99
Cerium	2.0±1.0	1.8±1.0	0.501	>0.99
Samarium	0.1±0.1	0.1±0.08	0.610	>0.99
Lead	49.0±23.2	38.2±26.3	0.097	>0.99
Phosphorus	288.6±227.8	256.9±205.1	0.574	>0.99
Sulphur	6011.8±2337.4	5653.0±4085.5	0.678	>0.99
Titanium	44.5±20.8	36.0±18.1	0.095	>0.99
Vanadium	6.74±5.9	4.3±3.6	0.058	>0.99
